# Job Insecurity and Innovative Work Behaviour: A Psychological Contract Perspective

**DOI:** 10.5334/pb.381

**Published:** 2018-01-04

**Authors:** Wendy Niesen, Anahí Van Hootegem, Tinne Vander Elst, Adalgisa Battistelli, Hans De Witte

**Affiliations:** 1 Thomas More, BE; 2 Work, Organisational, and Personnel Psychology Research Group, KU Leuven, BE; 3 IDEWE, External Service for Prevention and Protection at Work, BE; 4 EA4139 Laboratory of Psychology, University of Bordeaux, FR; 5 Optentia Research Focus Area, Vanderbijlpark Campus, North-West University, SA

**Keywords:** Innovative work behaviour, job insecurity, psychological contract breach, idea generation, idea implementation

## Abstract

Innovation is considered to be of crucial importance for organisational survival and growth, and in this respect employees play a leading role, as they are the ones who develop innovative ideas. At the same time, the struggle for organisational survival and growth gives rise to perceptions of job insecurity. To date, few studies have explored how employees’ innovative work behaviour (IWB) is influenced by the perceived threat of job loss (i.e. job insecurity). As both job insecurity and IWB are increasingly salient in light of organisational change and competition, the present study examines the relationship between job insecurity and IWB, as well as the role of psychological contract breach in explaining this relationship. We hypothesized a negative relation between job insecurity and innovative work behaviour, with psychological contract breach as a mediator in this relationship. Participants were 190 employees from an industrial organisation that had faced restructuring and downsizing for several years. Contrary to our predictions, no direct association was found between job insecurity and the two sub-dimensions of innovative work behaviour (i.e., idea generation and idea implementation). Indirect relationships, however, were found between job insecurity and the two types of IWB through psychological contract breach. Surprisingly, psychological contract breach was positively related to idea generation and idea implementation. These findings shed new light on the relationship between job insecurity and IWB.

## Introduction

Technological changes, the aftermath of the economic recession, globalization and worldwide competitiveness have caused organisations to resort to different kinds of restructurings, often in the form of employee downsizing (Burke & Ng, 2006). Although organisations employ these measures to increase productivity and improve their cost structures, research has shown that an organisation’s performance rather deteriorates than improves following downsizing (Datta, Guthrie, Basuil, & Pandey, 2010). These detrimental effects may be explained by increased perceptions of job insecurity during the restructuring as well as in the post-restructuring period. A review by Quinlan and Bohle (2009) on the effects of downsizing for employee well-being demonstrated that increased job insecurity explained the negative consequences of downsizing on health and safety in most of the reviewed studies. In addition, de Jong and colleagues (2016) reviewed longitudinal studies about the impact of restructuring on employee well-being. Similarly, their results indicated that experienced job insecurity was a key mechanism in interpreting the adverse outcomes of organizational restructuring.

The same societal and industrial changes that are responsible for increased organisational restructuring, and thus for heightened feelings of job insecurity, have also increased the importance of innovation for organizations’ competitiveness. In addition, many organisations expect that restructuring and downsizing will enhance innovation (Probst, Stewart, Gruys, & Tierney, 2007). While current changes in the labour market have inspired scholarly interest in job insecurity and employees’ innovative work behaviour (IWB) separately, the relationship between both concepts has remained under-researched. In line with West and Farr (1989), we define IWB as “the intentional introduction and application, within a role, group or organisation of ideas, processes, products or procedures, new to the relevant unit of adoption designed to significantly benefit the individual, the group, organisation or wider society” (p. 16). Prior research has demonstrated that these employee innovations are important drivers for an organization’s success and thus the security of its members (Janssen, 2000; Ma Prieto & Pilar Perez-Santana, 2014; De Spiegelaere, Van Gyes, De Witte, Niesen, & Van Hootegem, 2014). Since scholars argue that IWB consists of two sub-dimensions (i.e., idea generation and idea implementation), we separately take both types of IWB into account (Scott & Bruce, 1994; West, 2002). The current study focuses on the question if and why job insecurity and IWB are related. We investigate these relationships in the context of a downsizing organisation, thereby contributing to a better understanding of the reactions of employees who are in the midst of a restructuring organisation.

Organisational restructuring and downsizing have also altered employees’ perceptions of and reactions to the employment relationship (Zhao, Wayne, Glibkowski, & Bravo, 2007). As the violation of promises made during recruitment has been found to explain several of job insecurity’s attitudinal outcomes (De Cuyper & De Witte, 2006), and psychological contract fulfilment has been shown to be an antecedent of innovative behaviour (Ramamoorthy, Flood, Slattery, & Sardessai, 2005), the present study aims to examine whether breach of the psychological contract plays an explanatory role in the association between job insecurity and both dimensions of IWB.

This study contributes to the literature in the following ways. First, a new possible outcome of job insecurity is introduced, namely IWB. Second, we examine the concept of IWB more closely, by investigating the relationship between job insecurity and two different dimension of IWB, that is, idea generation and idea implementation. By doing so, we assist in resolving the controversy of the similarity of the antecedents of both stages. Finally, we aim to advance insights in the mechanism underlying the negative outcomes of job insecurity by analysing whether psychological contract breach mediates the relationship between job insecurity and IWB. This study might be considered as innovative since the links of both job insecurity and psychological contract breach with IWB are under-researched topics.

## Job insecurity

This article focuses on job insecurity, which is defined as “the subjectively perceived likelihood of involuntary job loss” (Sverke et al., 2002, p. 243). As a result, job-insecure employees find themselves in an undesired twilight zone between employment and unemployment. Not surprisingly, job insecurity was found to relate to multiple stress reactions, such as anxious feelings, depression, somatization and psychiatric symptoms (Boya, Demiral, Ergör, Akvardar, & De Witte, 2008; Meltzer et al., 2010). Concerning behavioural outcomes, job insecurity has been associated with work withdrawal behaviours (Probst, 2005), decreased OCB (Reisel, Probst, Chia, Maloles, & König, 2010) and exit behaviour of the best employees (Berntson, Näswall, & Sverke, 2010).

## Innovative work behaviour

Behaving innovatively at work refers to the intentional generation and implementation of new ideas at work in order to benefit role performance, group performance or the organisation in general (Janssen, 2000). IWB is a behaviour performed for the benefit of the organisation (Axtell, Holman, Unsworth, Wall, & Waterson, 2000; De Jong & Hartog, 2007). Several employee behaviours may help organisations to become more innovatively, and accordingly, IWB is considered “a construct that captures all behaviours through which employees can contribute to the innovation process” (De Jong & Hartog, 2007, p. 43). There is considerable evidence that organisations need to rely on the innovative abilities of all employees in order to become more innovative. Two phases are typically distinguished in the innovation process, namely the generation of ideas and subsequently the implementation of these ideas. Idea generation concerns the creation of ideas that are relatively new, that is, new in the context in which they will be implemented, and offer an improvement or solution to problems an employee has encountered. Idea generation is therefore similar to creativity, as both behaviours concern the rise of new ideas (West, 2002). Idea implementation refers to the adaptation and convergence of these ideas with daily work practices.

## Job insecurity and idea generation

According to West (2002), idea generation requires an environment which is undemanding, that is, an environment low in external demands, threats or uncertainty. Such an undemanding environment is unlikely to be present for insecure employees, since they perceive their environment as uncertain and threatening. Likewise, Pech (2001) assumed that downsizing in organisations hinders the creativity of employees, and thus their idea generation. Due to a lack of research on the association between job insecurity and idea generation, we rely on studies that focus on the relation between variables that are closely related to job insecurity and idea generation, such as restructuring and creativity, respectively. Probst and colleagues (2007) have, for instance, demonstrated a negative relationship between job insecurity and creativity, both in an experiment and a field setting. Additionally, Cascio (1993) found restructuring to lead to an increase in risk adverse thinking. Idea generation is likely to suffer from restructurings since the generation of a new idea always includes the risk of unsuccessfulness. Similar findings emerged from a study by Amabile and Conti (1999), which demonstrated that organisational downsizing negatively impacted the work environment for creativity. Based on the aforementioned empirical evidence, a negative relation between job insecurity and the first phase of IWB is expected, leading to the following hypothesis:

***Hypothesis 1a:*** *Job insecurity and idea generation are negatively related*.

## Job insecurity and idea implementation

For an innovative idea to improve organisational functioning or increase profit, the testing and commercialising of an idea (i.e., idea implementation) is crucial. In spite of the importance of this application-oriented behaviour and agreement on the effect of external factors on innovative efforts, research on the relation between job insecurity and idea implementation is scarce. Idea implementation might manifest itself in various ways, such as by persuading others of the value of the idea, by testing and adapting an idea or by modifying the workplace to the innovation (de Jong & Hartog, 2007). Difficulties often arise, such as an increase in conflicts with co-workers when engaging in IWB, explaining why only few innovations are truly implemented (Janssen, 2003). Idea implementation may therefore be considered as a behaviour that requires considerable effort from employees. When experiencing feelings of job insecurity, employees are less likely to engage in behaviours which require extra effort, as they tend to withdraw from the organisation. Withdrawal implies that employees disengage from their work and their organisation, resulting in lower levels of performance and effort (Abramis, 1984), as well as intentions to leave the organisation and apply for a job elsewhere (Cheng & Chan, 2008). Similarly, employees might withdraw from the insecure job situation by reducing the effortful, change-related behaviour of idea implementation.

As successful idea implementation requires sustained efforts, the negative association between job insecurity and employees’ efforts might provide guidance as to how job insecurity might relate to idea implementation. Already in 1984, Greenhalgh and Rosenblatt found a negative relationship between job insecurity and exerted effort, and a positive relationship between job insecurity and resistance to change. Brockner, Grover, Reed, and Dewitt (1992) demonstrated that high levels of job insecurity negatively relate to expended effort. The strongest evidence, however, comes from Bommer and Jalajas (1999), who found that the threat of organizational downsizing negatively relates to employees’ willingness to make innovative suggestions to supervisors. In keeping with the aforementioned theoretical and empirical arguments, we hypothesize that:

***Hypothesis 1b:*** *Job insecurity and idea implementation are negatively related*.

## Psychological contract breach as an explanatory variable

Apart from studying the direct relationship between job insecurity and both dimensions of IWB, this article further aims to analyse the process through which these variables are related, by including psychological contract breach as a mediational mechanism. Psychological contract breach derives from psychological contract theory (Rousseau, 1989), and is defined as “the idiosyncratic set of reciprocal expectations held by employees concerning their obligations and their entitlements” (McLean Parks, Kidder, & Gallagher, 1998, p. 698). These reciprocal obligations form the essence of the psychological contract (Rousseau & McLean Parks, 1993), and generally consist of contributions of the employee in terms of time, effort and work attitude, versus promised benefits on the part of the employer, such as job security, salary, appreciation, challenging work or prospects for promotion (Rousseau & McLean Parks, 1993). When one or both parties feel that the other party did not fulfil his/her promises, psychological contract breach occurs (Robinson & Morrison, 2000).

Prior research has demonstrated that organisational restructuring is significantly related to perceptions of psychological contract violations, mostly due to perceived broken promises regarding job security (Turnley & Feldman, 1998). Since the promise of job security is included in the traditional psychological contract, which is dominant in Europe (De Witte, 2005), employees might expect that when they fulfil their part of the deal, the organisation will reciprocate by offering job security (De Cuyper & De Witte, 2006). De Cuyper and De Witte (2006, 2007) empirically demonstrated that feelings of job insecurity were linked to psychological contract breach. Next, we may predict a negative relationship between psychological contract breach and IWB. When a fair exchange between employer and employee is lacking, employees will lower their innovative contributions to the organisation (IWB) (Robinson & Rousseau, 1994). These innovative efforts can be very diverse: an employee may help his/her organisation by introducing new ideas (i.e., idea generation) or by applying these ideas to the daily functioning of the organisation (i.e., idea implementation). Employees will invest less, as a way to no longer feel short-changed (Robinson, 1996) and to restore the equity between their investments or costs and the benefits they receive (Adams, 1965).

In the context of organizational downsizing, however, a reversed and positive relationship between psychological contract breach and IWB might also be possible. The study of Janssen (2002) demonstrated that employees scale back on their IWB when they perceive that their innovative efforts are under-rewarded by the organisation. In other words, employees expect to be rewarded for their innovative work behaviour. At the same time, prior research has shown that employees that have been affected by organisational restructuring report an imbalance between their exerted efforts and the rewards they get from their organisation (Tsutsumi, Nagami, Morimoto, & Matoba, 2002). Hence, engaging in IWB might not be sufficiently rewarded in a downsizing organization, thereby leading to a perceived breach of the psychological contract. This would entail a positive relationship in which IWB leads to perceptions of psychological contract breach.

Yet, the current study expects a negative relationship that flows from psychological contract breach to IWB, based on the aforementioned theoretical and empirical evidence which predominantly points to this negative pathway. These arguments align with the longitudinal study of Ng, Feldman, and Lam (2010) that demonstrated that the perception of psychological contract breach leads to lower levels of innovative behaviours. This negative effect was interpreted as a form of negative reciprocation and considered as a way to react to psychological contract breach.

In sum, job insecurity may be positively related to psychological contract breach, which in turn may be related to lower levels of IWB in terms of idea generation and idea implementation. This implies that psychological contract breach mediates the relationship between job insecurity and IWB. Prior studies have found psychological contract breach to account for the relationship between job insecurity and the behavioural outcome of self-rated performance, thereby offering indirect support for the expected mediating effect (De Cuyper & De Witte, 2006; King, 2000).

***Hypothesis 2:*** *Psychological contract breach mediates the relationship between job insecurity and IWB, i.e., idea generation (H2a) and idea implementation (H2b)*.

## Method

### Organizational context

This study was run in an industrial organisation in the region of Brussels that had recently undergone multiple restructurings and lay-offs. Due to the financial crisis, the organisation reduced almost 30% of their workforce in the two years prior to the data collection. At the time of data gathering, the organization was still facing restructuring. More specifically, two more branches were planned to close and some employees were going to be transferred to a French company that was linked to the downsizing company. One year after the data was collected, the organisation downsized another 20% of their remaining employees.

### Sample and procedure

Data were collected by means of an online questionnaire that was part of a larger scale study on employee well-being within the organization. White-collar workers were invited to fill out the questionnaire by mail, in which a link gave access to the online questionnaire. Blue-collar workers received an invitation by internal postal services with a personal access code. By use of this code, they could fill out the questionnaire on public computers provided by the company. The questionnaire was sent to 578 employees and was provided both in Dutch and in French; 203 employees completely filled out the questionnaire (response rate of 35%). We excluded 13 participants who were in a higher management position, as they were involved in the decision-making of the restructuring process, resulting in a final sample of 190 employees.

The sample consisted of 84.5% men (*n* = 163) and 15.5% women (*n* = 30). The average age of the respondents was 45.87 years (*SD* = 7.83), with ages ranging from 23 to 60 years. The mean tenure was over 2.18 years (*SD* = 9.08). The majority of the respondents (99.5%) had a permanent contract, and 88.6% worked on full-time basis. Our sample included 1.6% (*n* = 3) unskilled blue collar workers, 24.4% (*n* = 47) skilled blue-collar workers, 28.5% (*n* = 55) lower level white collar workers, 23.8% (*n* = 46) intermediate white collar workers, and 21.8% (*n* = 42) upper white collar workers/middle management. A total of 112 (58%) employees spoke Dutch, while 81 (42%) of the respondents spoke French.

### Measures

All measures were restricted to self-reports. Unless stated otherwise, all scales were found to have single-factor structures (PCA, Varimax rotation).

#### Job insecurity

Job insecurity (α = .88) was measured with four items of the Job Insecurity Scale (De Witte, 2000; Vander Elst, De Witte, & De Cuyper, 2014), with responses varying between 1 (*strongly disagree*) and 5 (*strongly agree*). A sample item is “I feel insecure about the future of my job”.

#### Innovative work behaviour (IWB)

IWB was measured using 10 items from de Jong and den Hartog (2010) that were rated on a five-point Likert scale ranging from 1 (*never*) to 5 (*always*). Principal component analysis revealed two factors with eigenvalues over one, explaining 68% of the variance. The first factor corresponded to the generation of ideas and the second factor matched idea implementation. This two-factor solution was preferred over the one-factor solution as it corresponds to the aforementioned theoretical distinction and is more easily interpretable. The first factor was labelled ‘idea generation’ (four items, α = .87), while the second was labelled ‘idea implementation’ (five items, α = .90). Sample items for the first and second scale are respectively “How often do you search out new working methods, techniques or instruments” and “How often do you contribute to the implementation of new ideas?”. One item was eliminated from the idea generation scale as it decreased the internal consistency of the scale considerably (from α = .83 to α = .87). Results will be reported separately for both dimensions.

#### Psychological contract breach

Psychological contract breach (α = .82) was measured with five items, with responses ranging from 1 (*completely disagree*) to 5 (*completely agree*) from Robinson and Morrison (2000). This measure assessed the overall evaluation of how well the employer has fulfilled the promises that were made during recruitment. A sample item is “Almost all the promises made by my employer during recruitment have been kept thus far” (reverse coded).

#### Control variables

Following the recommendations of Becker (2005), we included control variables that were likely to relate to the dependent variables. Organizational tenure (years) was included as a covariate since it negatively relates to IWB (Dorenbosch, Engen, & Verhagen, 2005). Education and occupational position are both related to IWB (Oldham & Cummings, 1996; Spreitzer, 1995). However, to avoid multi-collinearity, we included only one of them in the analyses. Occupational position was preferred over education. While the educational level of employees influences their potential to be innovative in general, occupational position can be considered as a situational facilitator or inhibitor that influences the extent to which an employee has the opportunity to be innovative in his/her job, thereby having a large influence on the actual innovative behaviour of an employee. We treated occupational position (1 = unskilled blue collar worker; 2 = skilled blue collar worker; 3 = lower level white collar worker; 4 = intermediate white collar worker; 5 = upper white collar worker/middle management/executive staff) as a continuous variable as these occupational positions represent a range going from less skilled to highly skilled (De Cuyper et al., 2014). In addition, we compared whether the results differed if the analyses were run with four dummy variables, which was not the case. Since male managers generally have a more positive attitude towards creativity (Mostafa, 2005) and are more innovation oriented (Mueller & Thomas, 2000), gender (1 = male; 0 = female) was also inserted as a control variable. In addition, fulltime employment (1 = fulltime employment; 0 = otherwise) was included as a covariate, because these employees spend more time at work, giving them more possibilities to be innovative. Finally, language (0 = French, 1 = Dutch) was also included. Note that we did not control for the possible effect of method (invitation by mail or internal postal services), as the method was dependent upon the occupational position of the employees (see above), which was already selected as a covariate.

### Analyses

The hypotheses were tested using the software package SPSS. Hypothesis 1a and 1b were tested by means of hierarchical regression analysis (HRA), whereas Hypothesis 2a and 2b were analysed with the PROCESS macro (model 4) (Hayes, 2013). PROCESS is a computational procedure that uses bootstrapping to test direct and indirect effects in mediation. This approach allows for more valid conclusions as normality is not required for the sampling distribution of the indirect effects (Mackinnon, Lockwood, Hoffman, West, & Sheets, 2002). Moreover, PROCESS allows to employ heteroscedasticity consistent standard errors to rule out heteroscedasticity in the model. All variables were standardised prior to analyses to produce standardised regression coefficients. The control variables were added to the model as covariates. Separate analyses were performed for both subscales of IWB (i.e., idea generation and idea implementation).

## Results

### Descriptive statistics

Table 1 displays the means, standard deviations and correlations among the variables. On average, the respondents scored moderately high on job insecurity (*M* = 3.34, *SD* = .94), indicating that they feel slightly insecure about the future of their job. As expected, job insecurity correlated positively with psychological contract breach. However, no relationship between job insecurity and both types of IWB was found. Surprisingly, psychological contract breach was positively correlated to idea generation as well as idea implementation. This suggests that employees who believe that their employer has not kept all his promises are more inventive and creative, and implement these ideas more often than employees who believe that their employer kept all of his promises.

**Table 1:** Summary of Means, Standard Deviations and Intercorrelations.

Variables	*M*	*SD*	1	2	3	4	5	6	7	8

1. Male	–	–								
2. Tenure	21.18	9.08	.10							
3. Fulltime	–		.16*	–.22**						
4. Occupational position	3.40	1.12	–.04	–.22**	.08					
5. Dutch	–	–	–.05	.03	–.11	–.03				
6. Job insecurity	3.36	.93	.15	.10	–.02	–.15*	.14			
7. PC Breach	3.55	.80	.04	.02	.02	–.06	.05	.33**		
8. Idea generation	3.43	.64	.07	–.24**	.12	.25**	.15*	–.01	.21**	
9. Idea implementation	2.86	.73	.19**	–.08	.20**	.18*	.06	.01	.16*	.67**

*Note.* *p < .05, **p < .01, ***p < .001.

### Hypothesis 1: Job insecurity and IWB

Hypothesis 1a concerned the negative relationship between job insecurity and idea generation. Contrary to our predictions, the HRA demonstrated that job insecurity did not significantly contribute in predicting this behaviour (see Table 2). Similarly, no significant link was found between job insecurity and idea implementation, thereby rejecting Hypothesis 1b (see Table 2). Hence, our data did not support Hypothesis 1.

**Table 2:** Hierarchical Regression Analyses Predicting Idea Generation and Implementation Behaviour from Job Insecurity and Breach of Psychological Contract.

	Idea Generation		Idea Implementation	
	β	ΔR^2^	β	ΔR^2^

Step 1		.13***		.11**
Male	.10		.18*	
Tenure	–.19**		–.03	
Fulltime	.05		.15*	
Occupational position	.21**		.18*	
Dutch	.17**		.09	
Step 2		.00		.00
Job insecurity	–.08		–.03	
Step 3		.05**		.03*
PC breach	.24**		.17*	
Adjusted R^2^		.15		.10
*N*		190		190

*Note.* *p < .05, **p < .01, ***p < .001.

### Hypothesis 2: The mediating role of psychological contract breach

**Figure 1:**
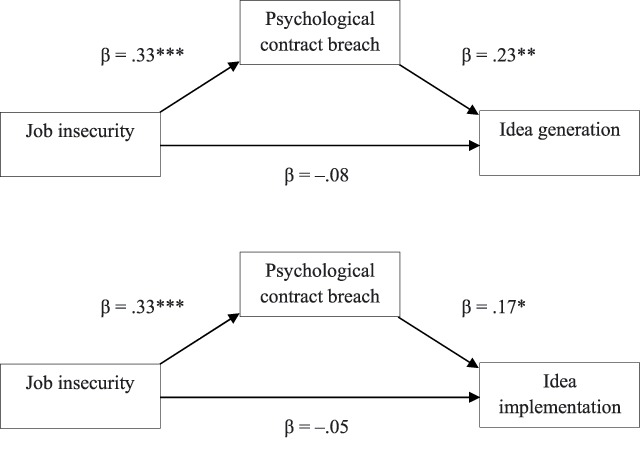
Mediation of psychological contract breach in the relation between job insecurity and innovative work behaviour. *Note*. *p < .05; **p < .01; ***p < .001.

Hypothesis 2a concerned the indirect relationship between job insecurity and idea generation through psychological contract breach. The mediation analysis showed a significant indirect effect of job insecurity on idea generation via psychological contract breach (effect = .05; 95% CIs [0.02, 0.11]) (i.e., the 95% CI did not include zero) (see Table 3), whereby job insecurity positively related to psychological contract breach, which in turn positively related to idea generation (see Figure 1). Contrary to our expectations, these results suggest that employees with a violated psychological contract display more acts of idea generation in comparison to employees whose psychological contract was not violated.

**Table 3:** Bootstrap Point Estimates and Bias-Corrected and – Accelerated (Bca) Confidence Intervals (Cis) for the Indirect Effects on Idea Generation and Idea Implementation.

Indirect effect	Effect	Boot SE	95% bias corrected and accelerated confidence interval	
			Lower	Upper

JI – PC breach – idea generation	.08	.03	.03	.16
JI – PC breach – idea implementation	.05	.03	.01	.13

*Note.* JI = job insecurity; SE= standard error.

Hypothesis 2b concerned the indirect relationship between job insecurity and idea implementation through psychological contract breach. The bootstrapping test provided evidence for psychological contract breach as a mediator in the relationship between job insecurity and idea implementation (effect = .04; 95% CIs [0.01, 0.10]). The results indicated that perceptions of job insecurity are associated with higher perceptions of psychological contract breach, which in turn is associated with higher levels of idea implementation. The positive relationship between psychological contract breach and idea implementation is in contract with what we hypothesized.

## Discussion

Organisations employ strategies of restructuring and downsizing with the aim of improving efficiency and competitiveness. Additionally, many organisations cite enhanced innovation as an expected outcome of the aforementioned measures (Probst et al., 2007). The present study investigated the relationship between job insecurity and innovative work behaviour (IWB), and the mediating role of psychological contract breach in this relationship. These constructs are especially relevant in the context of a downsizing organisation. Analyses were conducted separately for both dimensions of IWB, namely idea generating and idea implementation. The findings of the current study suggest that there is no direct relationship between job insecurity and both sub-dimensions of IWB. Our results did provide support for the mediating role of psychological contract breach in the relationship between job insecurity and IWB. We found a positive association between job insecurity and psychological contract breach. Contrary to our expectations, psychological contract breach was in turn positively related to idea generation and idea implementation. These results were confirmed by a bootstrapping analysis: psychological contract breach was found to explain the relationship between job insecurity and idea generation (H2a) and between job insecurity and idea implementation (H2b).

The absence of support for the direct relationship between job insecurity and idea generation (H1a) and idea implementation (H1b) could have several reasons. To start, the association between job insecurity and employees’ subsequent performance may depend on employees’ trust in the organisation, as trust has been found to impact the association between job insecurity and behaviour (Wong et al., 2005). Second, we might argue that the relationship between job insecurity and idea generation and idea implementation is not a linear relationship. This suggestion originates from Brockner and colleauges (1992) who found an inverted-U relationship between job insecurity and subsequent work effort. Finally, results suggest that innovative work behaviour could be considered as a more distal outcome of job insecurity, which implies that the association between job insecurity takes some time to develop or that this association is conditional upon other processes (Sverke et al., 2002).

In line with Hypothesis 2, our findings corroborated the mediating role of psychological contract breach in the relationship between job insecurity and IWB for both IWB components, that is, idea generation (H2a) and idea implementation (H2b). The present study thus extends existing knowledge on psychological contract breach as a potential mechanism behind the behaviour of job insecure employees. The positive relationship between psychological contract breach and idea generation and idea implementation was unexpected because it does not correspond to the literature on psychological contract breach that predicts negative effects for behavioural outcomes (Dulac, Coyle-shapiro, Henderson, & Wayne, 2008; Ng et al., 2010). These studies – typically conducted in a stable organizational environment – convey the common belief that the perception of psychological contract violation leads to a decrease in exerted efforts in order to restore balance in the employment relationship (Robinson & Rousseau, 1994).

However, a possible explanation for this positive relationship between psychological contract breach and IWB might lie in the specific context of organizational turmoil. More specifically, it might be that employees who have shown high IWB but are not adequately rewarded by their organisation experience this situation as a psychological contract breach. Building on the central assumption of psychological contract theory, which states that employees strive for a balance in their employment relationship, we might expect that employees increasingly expect their organisation to reciprocate the time and energy they have invested by behaving in an innovative way. When employees do not receive what they believe to be entitled to, namely high rewards in exchange for input in the form of IWB, they are likely to perceive a breach in their psychological contract. Other employees who invest less in the organisation may expect fewer incentives as they also have contributed less, resulting in the absence of psychological contract violation. This reasoning especially applies in the context of organizational downsizing, where employees have to perform highly innovative work, and where it is likely that they do not receive the expected rewards due to on-going restructuring and uncertainty. The imbalance between employees’ efforts to be innovative and the organisation’s reduced investments might cause employees to perceive a breach of the psychological contract breach. To date, however, no prior research has reported findings that are in line with our results. We conclude that, for idea generation and idea implementation, an indirect relationship with job insecurity through psychological contract breach was found.

Our findings contribute to research investigating the relationship between the changing work environment, which includes job insecurity, and employees’ IWB. By investigating psychological contract breach as an explanatory variable in our theoretical framework, we increased the understanding of the mechanism through which job insecurity is related to IWB. Our findings suggest that job insecurity and innovative behaviours are not directly related. Job insecurity, however, appears to indirectly relate to idea generation through psychological contract breach. Given the surprising results of this study, it may be advisable to wait until future studies replicate our results (i.e., a positive relationship between psychological contract breach and innovative work behaviour) before drawing practical implications. In general, we believe that the results of this study raise a number of questions which warrant further research.

### Limitations and future research

Several limitations concerning the study design and sample require further attention. First, all variables were measured through self-report questionnaires, which introduces a potential risk for common method bias (Doty & Glick, 1998). Despite this drawback, the subjective nature of job insecurity and psychological contract breach requires a self-report measure. Future studies, however, could benefit from including other-rated IWB. Second, because the present study is limited to cross-sectional data, no inferences about causality can be made (Mackinnon, Fairchild, & Fritz, 2007). A longitudinal follow-up study would allow to further examine the direction of the associations, thereby clarifying the debate about the direction of the relationships between psychological contract breach and innovative work behaviour. Another possibility for determining the causal impact of the different constructs, is to test the different relationships by means of an experimental design. The study of Probst and colleagues (2007), for instance, simulated the threat of being laid-off in a laboratory experiment. These studies, however, have the shortcoming of a lower ecological validity, as the artificially created conditions cannot fully capture the negative consequences and stressful nature of job insecurity.

Third, the current sample consisted of an organization that had recently undergone restructuring, which contributed to moderately high levels of job insecurity in our sample. As this survey was conducted in collaboration with the higher management, the low response rate of 34% might have been a reflection of decreased trust in the higher management. The generalizability of our findings are limited due to the absence of a representative sample size. Future research might benefit from replicating these findings in more representative samples.

The present study was particularly interested in IWB and how these behaviours relate to job insecurity and psychological contract breach. Nonetheless, the effects of job insecurity may also be explained by using other theoretical frameworks, such as Jahoda’s latent deprivation model (Selenko & Batinic, 2013) or a decrease in perceived control (Vander Elst, De Cuyper, & De Witte, 2011). Future research might benefit from examining how these theoretical constructs explain the relationship between job insecurity and IWB, and compare their relative importance. Another fruitful area for future research is to investigate the causal relationships between psychological contract breach and IWB. Previous research suggests that the effects of psychological contract breach might be subscribed to the effects of unmet expectations (Robinson, 1996) and/or to the damaged relationship and the decreased trust that it accompanies (Rousseau, 1989). The variable of trust may play an especially important role, as Wong and colleagues (2005) found that the effect of job insecurity on employees’ behaviour partly depends on this variable.

## Conclusion

The current study represents a first attempt to investigate the relationship between job insecurity and two sub-dimensions of innovative work behaviour (IWB), namely idea generation and idea implementation. While no direct relationship between job insecurity and both types of IWB was found, an indirect relationship between job insecurity and idea generation and between job insecurity and idea implementation through psychological contract breach was established. Contrary to our predictions, psychological contract breach was positively related to idea generation and idea implementation, indicating the importance of further theoretical and empirical research.

## Authors Information

Both Wendy Niesen and Anahí Van Hootegem are joint first authors.
